# Risk factors of lobar lymph node metastases in non-primary tumor-bearing lobes among the patients of non-small-cell lung cancer

**DOI:** 10.1371/journal.pone.0239281

**Published:** 2020-09-17

**Authors:** Jingwei Liu, Jian Li, Gang Lin, Zhiqiang Long, Qian Li, Bing Liu

**Affiliations:** 1 Department of Thoracic Surgery, Peking University First Hospital, Peking University, Beijing, China; 2 Department of Environmental Medicine and Public Health, Icahn School of Medicine at Mount Sinai, New York, New York, United States of America; 3 Department of Thoracic Surgery II, Key Laboratory of Carcinogenesis and Translational Research (Ministry of Education/Beijing), Peking University Cancer Hospital & Institute, Beijing, China; Baylor College of Medicine, UNITED STATES

## Abstract

**Purpose:**

Lobar lymph node metastases in non-primary tumor-bearing lobes (NTBL) are rarely reported. This study examined the risk factors of lobar lymph node metastasis in NTBL.

**Methods:**

We retrospectively studied 301 patients with non-small-cell lung cancer (NSCLC) who underwent surgical pulmonary resection with systematic lymph node dissection plus extended lobar lymph node dissection of NTBL. Patients were classified into positive and negative NTBL groups. Unconditional logistic regression was used to identify the risk factors for lobar lymph node metastasis in NTBL.

**Results:**

NTBL lobar lymph nodes were identified in 38 patients (12.6%). A higher proportion of adenocarcinomas occurred in the positive NTBL group compared to the negative NTBL group (73.7% vs. 46.4%, *P* = 0.01). Risk of NTBL lobar lymph node metastases was significantly elevated in the lower lobe of primary site compared to the upper lobe (OR = 2.61, 95% CI = 1.26–5.75, *P* = 0.01), and with adenocarcinomas compared to squamous cell carcinomas (OR = 2.75, 95% CI = 1.09–7.65, *P* = 0.04). No differences were observed when comparing left and right lobes. NTBL lobar lymph node metastasis was most often observed among patients with larger tumor size, N1/N2 nodal involvement, with lymph vascular invasion (LVI), and visceral pleural invasion (VPI).

**Conclusion:**

NTBL lobar lymph node metastases occurred more often in patients with a primary NSCLC tumor in the lower lobe, with adenocarcinomas, larger tumor size, N1/N2 nodal involvement, LVI or VPI. Extended lymphadenectomy including NTBL nodes may be clinically advantageous when these risk factors are present.

## Introduction

Lung cancer as the most common type of cancer worldwide, is of particular prominence in China where it ranks as the most common cause of cancer-related deaths [1]. Around 85% of lung cancers are non-small-cell lung cancer (NSCLC) [2]. While the incidence of NSCLC is decreasing in Western countries [3], it is increasing in developing countries such as in China [4]. The standard treatment for localized NSCLC involves surgical resection. However, due to its aggressive disease course, even early stage NSCLC after being radically resected commonly recurs, with a 5-year survival rate of approximately 60% for stage I and II of NSCLC [5].

Lymph node metastasis is a common pathological observation in NSCLC. Around 24% of stage I (T1 stage) NSCLC patients were diagnosed with lymph node involvement [6]. Nodal status is a major determinant of long-term survival of post-surgery NSCLC [7]. In patients eligible for surgical resection, identification of nodal metastases has formed the basis of prognostic stratification and implications for adjuvant therapy [8]. Surgical resection for stage I or II of NSCLC usually involves lobectomy (or pneumonectomy) with complete mediastinal lymph node dissection.

Most studies on lymphadenectomy have focused on systematic lymph node dissection, including N1 stations (levels 10–14) and N2 stations (levels 1–9) [9]. However, a series of pathologic examinations after pneumonectomy or bilobectomy have revealed the involvement of lobar lymph nodes at non-primary tumor-bearing lobes (NTBL) in some patients [10]. Enlargement of the lobar lymph nodes in the upper lobe has been often observed with a local recurrence in patients who have undergone a right lower lobectomy [11, 12]. All these findings imply that either aberrant lymphatic flow to the lobar lymph nodes of NTBL is preserved or it is secondary to overburdened lymph node involvement in certain patients with NSCLC. However, the lobar lymph nodes of NTBL are not included among those routinely resected according to the European Society of Thoracic Surgeons (ESTS) guidelines [13] and there have been only few reports [14, 15] on the prevalence and clinical features of lobar lymph node metastases of NTBL.

To provide more information on lobar lymph node metastases of NTBL, we retrospectively studied the clinicopathologic properties of this type of nodal involvement and the significance of extended NTBL lymph node dissection and evaluated the risk factors for lobar lymph node metastases of NTBL in NSCLC patients.

## Materials and methods

### Patients

**Fig 1:**
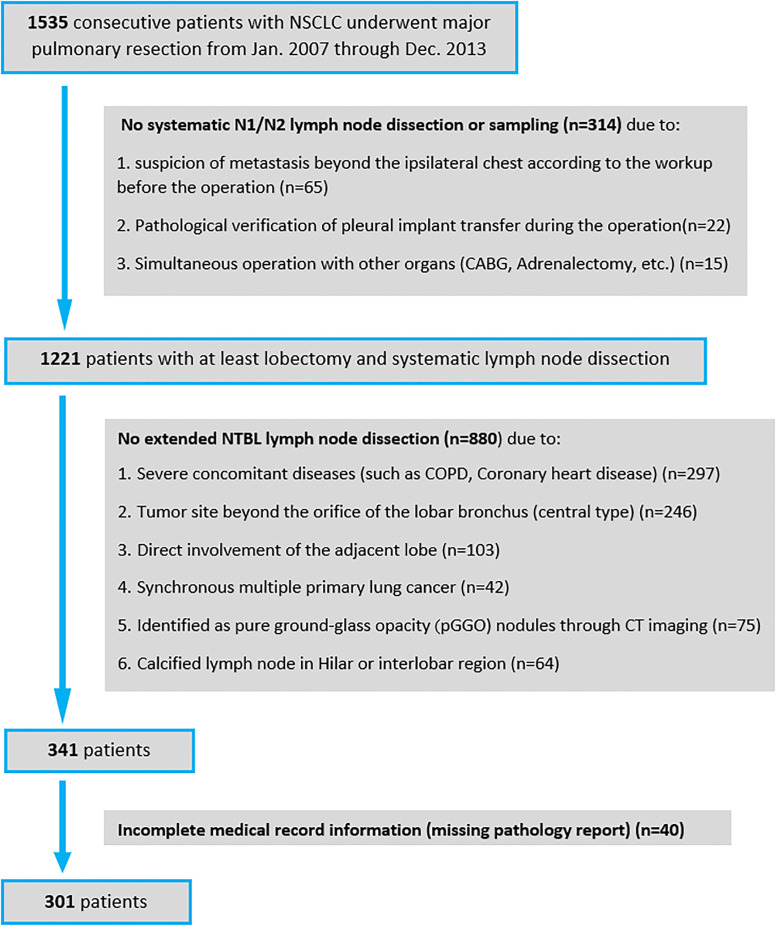
A CONSORT flow diagram of the study.

From January 2007 to December 2013, 1535 consecutive patients with NSCLC by pathological examination underwent major pulmonary resection in our institution. Among them, a total of 301 patients were included in the study according to the following criteria: (1) the primary tumor was located at the site peripheral to the orifice of the lobar bronchus; (2) there was no direct involvement of the adjacent lobe by the primary tumor; (3) the patient underwent surgical pulmonary resection (at least lobectomy was performed); (4) there was no synchronous multiple primary lung cancer. Details of the enrollment of the patients refer to the CONSORT diagram in Fig 1. Specifically, among 1535 patients, 314 patients were excluded since they had at least lobectomy but without systematic N1/N2 lymph node dissection or sampling; the other 1221 patients had at least lobectomy and systematic lymph node dissection. Among those 1221 patients, 880 patients were excluded since they didn’t have extended NTBL lymph node dissection due to the reasons listed in Fig 1. Please note that 75 cases of pure ground-glass opacity (GGO) nodules through imaging were excluded because of the small likelihood of lymphatic metastases [16]. Eventually a total of 301 patients were included in the study after 40 patients with incomplete records (missing pathology report) were also excluded. Of 301 patients who underwent major pulmonary resection with systematic N1/N2 and extended NTBL lobar lymph node dissection, lobectomy was performed on 203 patients, bilobectomy on 59 patients, and pneumonectomy on 39 patients. Written permission for extended lymph node dissection was obtained from every patient. Ethical approval was obtained from the Peking First Hospital ethics review board of biomedical research for patient data collection and storage in a secure database as well as reporting of outcomes. Informed consent was obtained from all individual participants included in the study.

### Preoperative investigations

Before surgery, all the patients underwent a contrast-enhanced chest computed tomography (CT) with high resolution. In addition, the serum tumor markers (carcinoembryonic antigen (CEA) squamous cell antigen (SCC), neuron-specific enolase (NSE), cytokeratin fragment (CYFRA 21–1), and cancer antigen (CA19-9), CT scan of the abdomen (or abdominal ultrasonography), cranium (or cranial magnetic resonance imaging) and whole-body bone scintigraphy were all conducted. Positron emission tomography-computed tomography (PET-CT) check was only performed in 61 patients (20.2%) due to the high cost and limited availability at the time of the study. Mediastinoscopy or EBUS were not routinely performed due to their invasive nature.

In the current study, NTBL lobar lymph node involvement was assessed with pathological staging since pathological staging is more meaningful than the clinical staging (clinical TNM staging information see S1 Table).

### Surgical procedures

**Fig 2:**
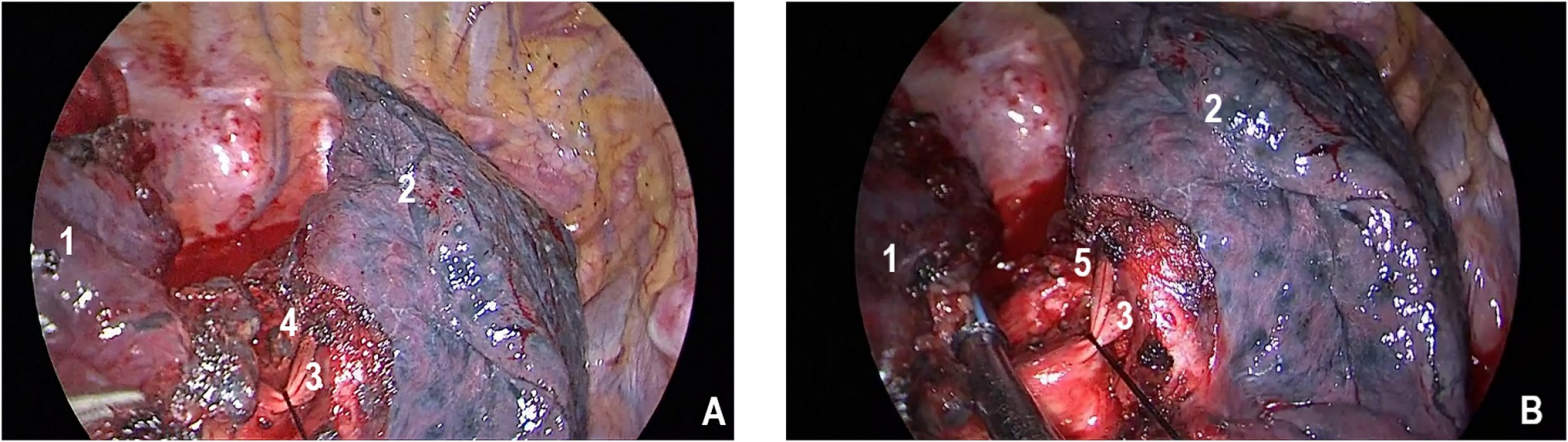
Intra-operative images of dissection of the lobar lymph node of the right upper lobe during a right lower lobectomy. A) The lobar lymph nodes of the right upper lobe were clearly seen with a silk thread passing around the ascending posterior branches. B) The root of the right upper lobar bronchus was explored after dissection of NTBL lobar lymph nodes. (1: right lower lobe; 2: right upper lobe; 3: ascending posterior branches of right pulmonary artery; 4: the lobar lymph nodes of the right upper lobe; 5: the root of the right upper lobar bronchus).

All patients underwent anatomic resection (lobectomy, bilobectomy and pheumonectomy) with systematic N1/N2 and extended NTBL lobar lymph node dissection. The extent of systematic lymphadenectomy (N1/N2) included dissection of lymph node stations 4 to 14 for left lung cancers, and dissection of lymph node stations 2 to 4, and 7 to 14 for right lung cancers. The extended lymphadenectomy in NTBL was performed behind the branches of the pulmonary artery and around the non-primary lobar bronchi, with the root of the adjacent lobar bronchus explored after the extended dissection. On the right side, the dissection of lobar lymph nodes in NTBL were targeted on the lower lobe if the lobectomy of middle lobe was performed; the dissection were targeted mostly on the middle lobe if the lobectomy of upper lobe was performed; the lymph nodes in either middle or upper lobes were dissected due to the suspected interlobar lymph nodes of adjacent lobe if the lobectomy of lower lobe was performed. All the resections were R0. As shown in Fig 2, two representative intra-operative images indicate the extend of lymphadenectomy. As of 2012, resections were performed using the video assisted thoracic surgery (VATS). In total, open thoracotomy was performed on 242 patients and VATS was performed on 59 patients.

Resection was considered complete when there was no microscopic or macroscopic tumor at the bronchial or vascular margins and no macroscopic residual disease in nodal areas. Resected lymph nodes other than NTBL lobar lymph nodes were defined by following the International Association for the Study of Lung Cancer Staging. All patients underwent staging [17] to determine a final pathologic stage (pTNM). the patients of stage II and IIIA received adjuvant chemotherapy following the National Comprehensive Cancer Network (NCCN) guidelines.

### Clinical data collection and pathological findings

The baseline characteristics of the patients, including gender, age, smoking history, tumor location, histologic type, size of tumor, lymph node metastases, lymphovascular invasion (LVI) and visceral pleural invasion (VPI), were collected from the medical record system of the Peking First Hospital.

Resected tumor lesion and dissected lymph nodes were examined for pathological diagnosis at our pathology department. Histopathologic analysis was performed by following the 2015 World Health Organization classification of tumors of the lung, pleura, thymus, and heart [18]. LVI was defined as unequivocal presence of floating tumor cells in lymphatic vessels (lack of supporting smooth muscles or elastic fibers in the vessel wall) or blood vessels. VPI was defined as tumor cells beyond the elastic layer of the visceral pleura, regardless of tumor exposure on the pleural surface.

Primary tumors were classified into squamous cell carcinoma (SCC), adenocarcinoma (ADC), and other types of NSCLC, in accordance with the World Health Organization International Histologic Classification of Tumors [18]. The tumor size was divided into 4 groups (≤ 3 cm, 3 ~ ≤ 5 cm, 5 ~≤ 7 cm and > 7 cm) in accordance with the 8^th^ Tumor Node Metastasis (TNM) classification for Lung Cancer [19].

### Statistical analysis

Categorical variables were expressed as frequencies and percentages, and comparisons of ratios were performed using the chi-square test or Fisher’s exact test. We ran several separate logistic regression models with a limited number of predictor variables based on prior literatures [20]. There was only one clinical predictor adjusted by one confounding variable (smoking status) in the final model. Adjustments for age and gender did not result in material changes for the observed associations and thus were not included in the final model. Unconditional logistic regression was used to estimate the association between clinical variables (primary tumor location, histologic type, tumor size, lymph nodes metastases, VPI, and LVI) and the risk of metastasis in NTBL. Potential confounders were selected based on prior knowledge as well as a 10% change-in-estimate criterion [20]. Based on these criteria, the final model was adjusted by smoking status (yes vs. no). Adjustments for age and gender did not result in material changes for the observed associations and thus were not included in the final model. Odds ratios (ORs) and 95% confidence intervals (CIs) were calculated to estimate the strength of the association and the precision of the estimates. All analysis was conducted using R statistical software (R version 1.0.143). All statistical tests were two-sided, and *P* < 0.05 was considered statistically significant.

## Results

### Demographic and clinical characteristics of the study participants

A total of 301 NSCLC patients were included in this study. Patients were classified into two groups according to whether the NTBL lobar lymph node was found to be involved after pathological examination. There were 38 patients (12.62%) in the positive NTBL group and 263 (87.38%) in the negative NTBL group. There are 97.3% patients (37 out of 38) with positive lymph nodes (N1 and N2) proven by final pathology in the positive NTBL group, which is much significantly higher than that in the negative NTBL group (134 out of 263, 51%, *P* < 0.001). The positive rate of N2 was 89.4% (34/38) in the positive NTBL group and 30.8% (81/263) in the negative NTBL group. The clinicopathological characteristics of the groups are listed in Table 1. The two groups were well balanced in terms of age and gender, but there was a significantly higher percentage of smokers in the negative NTBL group compared to the positive NTBL group (*P* = 0.01). Therefore, smoking history was controlled for in all subsequent analyses. There were also significant differences between the groups in terms of primary tumor location (*P* = 0.04) and histological type (*P* = 0.01). The most common location of the primary tumor was the right lower lobe (12 out of 38, 31.58%) in the positive NTBL group, while the most common primary tumor location in the negative NTBL group was the right upper lobe (88 out of 263, 33.46%). A significantly higher proportion of adenocarcinoma cancer was found in the positive NTBL group compared to the negative NTBL group (73.68% vs. 46.39%, *P* = 0.01).

**Table 1:** Clinicopathologic characteristics among NSCLC patients stratified by NTBL status.

Characteristics	NTBL (-) (N = 263)		NTBL (+) (N = 38)		*P* value
	N	%	N	%	
Gender					0.15
Male	180	68.44	21	55.26	
Female	83	31.56	17	44.74	
Age (years)					0.68
≤ 50	41	15.59	8	21.05	
≤ 60	78	29.66	10	26.32	
> 60	144	54.75	20	52.63	
Smoking history					0.01
No	106	40.30	24	63.16	
Yes	157	59.70	14	36.84	
Primary tumor location					0.04
LLL	38	14.45	11	28.95	
LUL	47	17.87	2	5.26	
RLL	74	28.14	12	31.58	
RML	16	6.08	4	10.53	
RUL	88	33.46	9	23.68	
Histologic type					0.01
AD	122	46.39	28	73.68	
SCC	109	41.44	7	18.42	
Others*	32	12.17	3	7.89	
Tumor size (cm)					0.27
≤ 3	108	41.06	10	26.32	
3 ~ ≤ 5	90	34.22	15	39.47	
5 ~ ≤ 7	45	17.11	10	26.32	
> 7	20	7.60	3	7.89	
Lymph nodes metastases					< 0.001
N0	129	49.0	1	2.7	
N1	53	20.2	3	7.9	
N2	81	30.8	34	89.4	
VPI					0.02
No	192	73.00	20	52.63	
Yes	71	27.00	18	47.37	
LVI					0.08
No	210	79.85	25	65.79	
Yes	53	20.15	13	34.21	

LLL, left lower lobe; LUL, left upper lobe; LVI, lymphovascular invasion; NTBL, non-primary tumor-bearing lobe; RLL, right lower lobe; RML, right middle lobe; RUL, right upper lobe; TBL, tumor-bearing lobe; VPI, visceral pleural invasion.

*Others including large cell carcinoma, adenosquamous carcinoma, atypical carcinoid, carcinoma of salivary gland, etc.

Totally 8678 lymph nodes were dissected from 301 patients. Based on anatomical locations, they are categorized into 3 groups: from N1 region (including stations 10, 11 & 12), from N2 region (including stations 1–9, mediastinal lymph nodes), and from NBTL region. Number range, mean number and average number of dissected lymph nodes in each category have been shown in S3 Table, based on their NTBL positive and negative.

The VATS resections were performed on 59 patients since 2012, including 51 cases of NTBL negative and 8 cases of NTBL positive. Thoracotomy was performed on 212 cases of NTBL negative and 30 cases of NTBL positive. There is no significant difference between them (P = 0.530) (S4 Table).

### Distribution of lymph node metastases in 38 positive NTBL patients

As shown in Table 2, of the 38 positive NTBL patients, interlobar, hilar, and mediastinal lymph node metastases were all observed in the majority. Of the 25 patients with right-sided malignancy, interlobar and mediastinal lymph node metastases were observed in the majority. The mediastinal metastasis was observed in 12 out of 13 patients with left-sided malignancy.

**Table 2:** Distribution of lymph node metastases in 38 positive NTBL patients.

Lymph Node Locations	Primary Site				
	RUL (n = 9)	RML (n = 4)	RLL (n = 12)	LUL (n = 2)	LLL (n = 11)
Lobar					
Node 12 upper	9	0	8*	2	11
Node 12 middle	5	4	6*		
Node 12 lower	4	4	10	2	9
Interlobar (node 11)	8	3	6	1	6
Hilar (node 10)	5	2	4	1	5
Mediastinal (nodes 1–9)	8	4	10	2	10

LLL, left lower lobe; LUL, left upper lobe; RLL, right lower lobe; RML, right middle lobe; RUL, right upper lobe; NTBL, non-primary tumor-bearing lobe.

*Both patients had lobar lymph node metastases around the middle lobe bronchus.

As shown in the Table 3, 38 cases of NTBL positive patients were categorized into four groups based on conventional N staging of lung carcinoma. Indeed, of the 38 NTBL-positive patients, 34 were N2 (ipsilateral mediastinal lymph node metastasis) and 3 were N1 (hilar and/or interlobar lymph node metastasis). 34 cases of N2 included 31 cases of non-skip N2 and 3 cases of skip N2. These 38 NTBL positive patients had NTBL metastasis. N staging was performed accordingly. Since conventional TNM staging doesn’t specify whether NTBL lymph nodes belong to N1, we just temporarily classified it as N1. Only one patient had no lymph node metastasis, but with NTBL metastasis. Therefore, its N stage needs to be adjusted from N0 to N1. The 3 patients with N2 had false skipped metastasis, which indicates that NTBL metastasis would fill up the missing metastasis information in the N1 area. In 3 cases of N1, the single-station N1 was changed to multi-station N1, which refines the N stage of the patient and helps judge the prognosis and guide adjuvant therapy. In summary, NTBL dissections have detailed the clinical stages of those 7/38 patients.

**Table 3:** Staging of lymph node metastases in 38 positive NTBL patients.

	N1	N2	NTBL	Staging without considering NTBL	Staging with considering NTBL	
31 cases	+	+	+	N2	N2.	NTBL doesn’t affect staging
3 cases	+	-	+	Single-station N1	multi-station N1	NTBL (+) indicates multi-station lymph node metastasis in N1 region, which helps with more accurate staging.
						Single-station N1 has a better prognosis than multiple-station N1.
3 cases	-	+	+	Skip N2	Non-skip N2	Skip N2 indicate that cancer “skips” over the N1 bronchopulmonary or hilar station to N2 ipsilateral mediastinal metastasis with longer survival and better prognosis than non-skip N2. NTBL (+) changes it from Skip N2 to N1+N2, Therefore, NTBL helps with more accurate staging.
1 case	-	-	+	N0	N1	NTBL helps with more accurate staging.

### Multivariable logistic regression analyses for risk of NTBL metastases

There were significant associations between the risks of NTBL metastasis and several clinicopathological factors (Table 4). Primary tumors located in the left upper (OR = 0.14, 95% CI: 0.02–0.59, *P* = 0.02) and right upper lobe (OR = 0.33, 95% CI: 0.12–0.88, *P* = 0.03) had a significantly reduced risk of NTBL metastasis compared to those located in the left lower lobe. The risk of NTBL metastases was significantly increased with adenocarcinomas compared to squamous cell carcinomas (OR = 2.75, 95% CI = 1.09–7.65, *P* = 0.04) but the increased risk was not significant in other histologic types of primary tumor (OR = 1.29, 95% CI = 0.26–5.03, *P* = 0.73). A significant trend was also observed between the tumor size and the risk of NTBL metastasis (OR = 1.48, 95% CI = 1.02–2.14, *P* = 0.04). As expected, all established factors related to lymph node metastases (lobar, interlobar/hilar, mediastinal and multi-station mediastinal) were also significantly associated with the risk of NTBL metastasis. VPI (OR = 2.65, 95% CI = 1.30–5.40, *P* = 0.01) and LVI (OR = 2.43, 95% CI = 1.11–5.16, *P* = 0.02), representative characteristics of tumor invasion, were also correlated with the risk of lobar lymph node metastases of NTBL.

**Table 4:** Multivariable logistic regression analyses evaluating risk factors of non-primary tumor-bearing lobe metastases.

Characteristics	NTBL (-)	NTBL (+)	Adjusted OR*	95% CI	*P* value
Location					
LLL	38	11	1.00		
LUL	47	2	0.14	0.02–0.59	0.02
RLL	74	12	0.53	0.21–1.35	0.18
RML	16	4	0.79	0.19–2.77	0.72
RUL	88	9	0.33	0.12–0.88	0.03
Histologic type					
Squamous cell carcinoma	109	7	1.00		
Adenocarcinoma	122	28	2.75	1.09–7.65	0.04
Others^#^	32	3	1.29	0.26–5.03	0.73
Tumor size (cm)					
≤ 3	108	10	1.00		
3 ~ ≤ 5	90	15	2.07	0.88–5.06	0.10
5 ~ ≤ 7	45	10	2.88	1.09–7.70	0.03
> 7	20	3	2.49	0.50–9.64	0.21
Trend			1.48	1.02–2.14	0.04
Lymph node metastases					
TBL lobar					
No	160	4	1.00		
Yes	103	34	16.06	6.04–56.0	<0.001
Interlobar/hilar					
No	230	12	1.00		
Yes	33	26	16.45	7.54–38.15	<0.001
Mediastinal					
No	182	4	1.00		
Yes	81	34	21.67	8.14–75.48	<0.001
Multi-station mediastinal					
No	240	13	1.00		
Yes	23	25	20.84	9.39–48.97	<0.001
VPI					
No	192	20	1.00		
Yes	71	18	2.65	1.30–5.40	0.01
LVI					
No	210	25	1.00		
Yes	53	13	2.43	1.11–5.16	0.02

AD, adenocarcinoma; LLL, left lower lobe; LUL, left upper lobe; LVI, lymph avascular invasion; NTBL, non-primary tumor-bearing lobe; RLL, right lower lobe; RML, right middle lobe; RUL, right upper lobe; TBL, tumor-bearing lobe; SCC, squamous cell carcinoma; VPI, visceral pleural invasion.

*: adjusted by the smoking history.

^#^Others including large cell carcinoma, adenosquamous carcinoma, atypical carcinoid, carcinoma of salivary gland, etc.

In addition, the risk of NTBL metastases was not associated with the right/left of the primary site. However, increased NTBL risk was observed in the lower primary site (OR = 2.61, 95% CI = 1.26–5.75, *P* = 0.01) compared to the upper site (Table 5). All analyses were additionally evaluated stratified by smoking status, but the results did not change significantly (S2 Table).

**Table 5:** Associations between primary site locations and NTBL metastases.

	NTBL (-)	NTBL (+)	Adjusted OR*	95% CI	*P* value
Location—1					
RLL/RML/RUL (right)	178	25	1.00		
LLL/LUL (left)	85	13	1.15	0.54–2.34	0.71
Location—2					
LUL/RUL (upper)	135	11	1.00		
LLL/RLL/RML (lower)	128	27	2.61	1.26–5.74	0.01

LLL, left lower lobe; LUL, left upper lobe; RLL, right lower lobe; RML, right middle lobe; RUL, right upper lobe; NTBL, non-primary tumor-bearing lobe.

*: adjusted by the smoking history.

## Discussion

This study retrospectively investigated the prevalence and risk factors of lobar lymph node metastasis from NTBL in patients with NSCLC. In our study, NTBL lobar lymph nodes were involved in 12.6% of patients, which is consistent with the published results, ranging from 6.5 to 13.2% [11, 14, 15, 21]. A higher proportion of adenocarcinomas were observed in the positive NTBL group compared to the negative NTBL group. NTBL lobar lymph node metastases occurred more often in patients with a primary NSCLC tumor in the lower lobe, with larger tumor size, N1/N2 nodal involvement, LVI or VPI. These results suggest that clinicians should consider extended lymphadenectomy that includes NTBL lobar lymph nodes when these risk factors are present.

NTBL is not a new concept, but there are quite a few studies involving lymph node dissection in this field. Yamanaka et al. suggests that NTBL metastasis is more common in primary right tumors and metastatic lymph node metastases, regardless of tumor sizes and histological types [14]; Li et al. suggests that NTBL metastases are common among pleural invaders, especially those whose tumors break through the pleura and invade adjacent lung tissue [21]. In addition, tumors located in the dorsal segment of the lower lobe of the lung and invading the posterior segment of the upper lobe are also prone to metastatic bronchial lymph node metastasis, and tumor size, N1/N2 lymph node status and NTBL lymph node metastasis seem to have little relationship; Sato et al. only described the necessity of dissecting the bronchial lymph nodes of the upper lobe during primary tumor located in the lower or middle lobe from a small sample (14 cases), and no risk factors for NTBL lymph node metastasis were involved [11]. To sum up, few studies have so far focused on NTBL metastasis and risk factors. Studies by Yamanaka et al. and Sato et al. focus on the research of NTBL metastasis with different mechanisms [11, 14]. Our study considers lymph channel blockage which leads to abnormal drainage. Li et al. indicates that the tumor invades the adjacent lung tissue and metastasizes to the adjacent intra-pulmonary lymph node through the subpleural lymphatic duct [21]. Our study has many cases of NTBL dissection, and systematically studied multiple risk factors including tumor size and location, pathological type, N1/N2 lymph node status, viceral pleural invasion and lymphatic vessel invasion and other clinical indicators which is different from the previous reports.

The basic pattern of pulmonary lymphatic flow of lung cancer is well understood to be from the primary tumor to lobar lymph nodes in the tumor bearing lobe and then interlobar and hilar; eventually reaching the ipsilateral and contralateral mediastinum [22, 23]. Such knowledge of anatomical lymphatic drainage pathways and metastatic spread patterns of lung cancer forms the basis for the N classification for the staging of lung cancer, providing practical guidelines for thoracic surgeons. However, locoregional N1 recurrence and the poor prognosis of lung cancer implied that aberrant lymph node metastases might occur, which was shown by metastases in the lobar lymph nodes of NTBL. Sakairi et al. [24], Nomori et al. [25] and Topol et al. [26] reported segmental lymph node involvement of non-primary tumor-bearing segments was found in some patients with segmentectomy, though the segmental lymph nodes metastases of tumor-bearing segment was fundamental. From an analogous viewpoint, we deduced that the frequency of adjacent lobar lymph node involvement might be higher than expected.

In this study, these so-called aberrant lymph node metastases to NTBL were observed in 38 of 301 patients (12.6%). This rate is higher than two previously reported studies: 6.5% by Yamanaka and colleagues [14] and 9.2% by Sato and colleagues [11]. But it is similar as the rate reported by Li and colleagues at 13.2% [15], and 12.7% [21]. These rates of anomalous lymph node metastases, which have received little investigation previously, suggest that extended lymphadenectomy including the lobar lymph nodes of NTBL should be considered to improve the accuracy of staging, and decrease locoregional recurrence.

In the present study, 34 out of 38 patients (89.4%) with lobar lymph node metastases of NTBL showed lobar lymph node involvement of the tumor-bearing lobe (TBL). Similar involvement frequency was seen in the mediastinal lymph node metastases. Moreover, the prevalence of hilar (node station 10) and interlobar (node station 11) lymph node metastases was significant higher in this group. Therefore, it is presumed that this kind of aberrant lymph node metastases are attributable to retrograde spread, which is usually caused by obstruction of lymphatic flow [27, 28]. We inferred that widespread proximal lymph node metastases probably induced increased collateral lymphatic spread to the lobar lymph nodes of adjacent lobes. That is to say, horizontal lymphatic spread to adjacent lobar lymph nodes would become more frequent when vertical spread was extensive. In addition to this rationale of retrograde spread, it is proposed that other possible patterns of lymphatic spread to NTBL lobar lymph nodes might exist, through an incompletely formed interlobar space or through subpleural lymphatics [29]. This hypothesis was first presented by Riquet M [30, 31] who reported that direct lymph vessels to lymph nodes located at the origin of the upper lobar bronchi were observed when dyes were injected into the subpleural lymphaticus of the basal segments. Moreover, from multivariable logistic regression analysis it was observed that tumor location and histological features were related to the risk of NTBL metastases. There was a reduced risk of NTBL metastases if primary tumors were located in the upper lobes compared to those located in the lower lobes. Meanwhile, adenocarcinoma might be associated with the high risk of NTBL metastases, and the risk was significantly reduced in the other histologic types of primary tumor (including large cell carcinoma, adenosquamous carcinoma, atypical carcinoid, or carcinoma of the salivary gland). Hence, histological features may also have a significant influence on the extended metastases in addition to the overburdened vertical spread of lymphatics.

Additionally, the elevated risk of NTBL metastases was observed predominantly with larger tumor size, with interlobar/hilum/N2 lymph node metastases, and with positive LVI and VPI. Therefore, we believe that the lobar lymph node in the TBL was frequently involved despite the presence of metastases to hilar or mediastinal nodes. As an important point of lymphatic flow between the intra-lobe and extra-lobe, lobar lymph nodes in TBL were mostly involved and considered to be the possible origin of lobar lymph node metastases in NTBL when the above-noted risk factors existed. Therefore, we recommended that complete dissection both of the lobar lymph node in TBL and NTBL be performed to improve the local control of patients with NSCLC. This suggestion is also supported by previous studies in patients with early stage NSCLC that have suggested similar risk factors for nodal and distant recurrence such as LVI in stage I [32] or tumor size, tumor differentiation, pleural invasion and bronchus invasion in stage I-IIA [33] so that complete lymph node dissection should be considered.

The LVI and VPI were also significantly associated with the risk of NTBL metastasis in the present study. Both LVI and VPI have been demonstrated to be the negative prognostic factors for the survival outcome in NSCLC in many studies [34–39]. The reason for the proportion of NTBL metastasis being significantly higher in patients with LVI or VPI remains unclear. However, lymphatic vessels are regarded as the important route by which neoplastic cells reach local and distant lymph nodes [40]. The visceral pleural is very rich in lymphatic vessels with an intercommunicating network arranged over the lung surface that penetrates the lung parenchyma to join the bronchial lymph vessels with drainage to various hilar lymph nodes [34]. To better understand the roles of LVI and VPI in NTBL metastasis, further study is necessary.

Although it remains controversial whether patients with lobar lymph node metastases in NTBL should undergo lobectomy/bilobectomy or pneumonectomy, our surgical procedure with extended lymphadenectomy has advantages for efficaciously removing the same lymph nodes as performed in pneumonectomy and preserving enough pulmonary function, especially on the right side [11, 14]. If technically feasible, pneumonectomy should be cautious due to high occurrence of postoperative complications, poor quality of life, cardiopulmonary dysfunction and long-term complications, especially on the right side. For patients with N1 involvement, similar results were reported in the studies on the metastases of the interlobar lymph nodes, favoring lobectomy with complete interlobar lymph nodes dissection instead of radical pneumonectomy [41–43]. Radical pneumonectomy remains the standard procedure for enlarged, bulky N1 nodes that have infiltrated the main bronchus (or main pulmonary artery).

240 patients were missing accurate pre-operative diagnosis with performing PET-CT scan and mediastinoscopy, but 88 patients were post-surgically confirmed as N2 positive. This group of unexpected mediastinal involvement disease is mostly a single-station mediastinal lymph node metastasis. Some literatures support single-station N2 metastasis as the first choice for surgical treatment [44–46]. Therefore, it is clinically feasible for this group of patients to continue to perform surgery and receive postoperative adjuvant chemotherapy. For unexpected multi-station mediastinal involvement, in the future, it is true that PET-CT or EBUS will be required to strengthen the preoperative accurate staging. Besides, the first choice for multi-station mediastinal metastasis is neoadjuvant chemotherapy before surgical treatment. with increased investment on medical equipment configuration, PET-CT and EBUS have become popular in our center. Therefore, N2 (+) patients will receive systematic preoperative evaluation through multidisciplinary teams (MDT). Only a small number of them with clinical manifestations, such as single station, diameter <1cm, normal CEA and other tumor markers level, would have surgical treatment as their first choice.

The limitations of the present study should also be addressed. Given the retrospective design, some selection bias could not be avoided: the percentage of metastatic N1 and N2 patients was more than that of other studies [11, 14]; this retrospective study was carried out in a single center with a limited number of enrolled patients. But the rate of aberrant lymph node metastases to NTBL was 12.6% (38 out of 301 patients), which is consistent with the published results, ranging from 6.5% to 13.2% [11, 14, 15, 21]. Besides, there is no significant difference between 301 enrolled patients with the excluded 40 patients who performed extended NTBL lymph node dissection but missed pathology diagnosis, in terms of demographic information such as sex and age (S5 Table). Another limitation is only 20% of patients had a preoperative PET-CT check due to the high cost and limited availability of this technique at the time of the study, and mediastinoscopy or EBUS were not routinely performed due to their invasive nature and high cost. We didn’t see the distribution difference of PET-CT scan between the NTBL positive (4 out of 38 cases) and negative groups (57 out of 263 cases) (P = 0.167) (S6 Table). Third, operations before 2011 were mostly performed with open thoracotomy while starting from 2011 video assisted thoracic surgery (VATS) was applied. But there is no significant difference between their percentage in consideration of NTBL negative and positive compositions (P = 0.530) (S4 Table). Fourth, the lobar lymph node metastases of NTBL is beyond the International Association for the Study of Lung Cancer (IASLC) lymph node map and the point of extended lymphadenectomy should be verified by providing meaningful data on prognostic differences, such as local recurrence rate, progression-free survival (PFS) and overall survival (OS). Fifth, adenocarcinoma cases were not classified into different subtypes since only 69 enrolled patients had pathological diagnosis following the 2011 lung adenocarcinoma classification. Since the percentages of all 5 subtypes of adenocarcinoma is low (S7 Table), for statistics and data consistency, we would like to continue following the 2004 WHO adenocarcinoma classification. Besides, among 35 cases classified as histological “others” in Table 4, 24 were large cell carcinoma, 5 were adenosquamous cell carcinoma, and the other 6 of atypical carcinoid, salivary gland-derived, and sarcomatoid carcinoma (S8 Table). The first two types (large cell carcinoma and adenosquamous cell carcinoma) do have a poor prognosis, but mainly shown as distant metastases, such as bone and brain metastases [47–50]. This study mainly focuses on the risk factors of lymph node metastasis. In the absence of evidence that large cell carcinoma and adenosquamous carcinoma are more prone to lymph node involvement than other rare histological types, current grouping in this study should be considered. In addition, standards of treatment are constantly evolving. With the latest guidelines, for some of the patients of this study, neoadjuvant chemo- and/or radiotherapy would be recommended. However, for the period of the reported study we took into account other reports (see for example [45, 46]) which suggest that for cases with small primary tumor and absence of multi-station metastases, additional treatment may be omitted. For T3/T4 cases with N0/N1 status, we concluded that surgery was sufficient without preliminary treatment. Thereupon, a future prospective study is required both on incidence of lobar lymph node metastases and the prognostic meaning of such extended lymphadenectomy. The current study was focused on the risk of metastases. Additional outcomes (such as after-surgery complications and long-term survival) present further important aspects and will be evaluated in future studies.

This study confirmed that lobar lymph node metastasis of NTBL occurs frequently in patients with NSCLC. We inferred that these extended lymph node metastases were mainly secondary to lymphatic congestion. In addition, we demonstrated that larger tumor size, N1 and N2 status, VPI, and LVI were independent risk factors for the involvement of the NTBL lobar lymph nodes. Thus, extended lymphadenectomy may be advantageous to control loco-regional recurrence and provide more accurate staging among patients with these risk factors.

## Supporting information

S1 Table Clinical staging information among NSCLC patients stratified by NTBL status. (DOCX)

S2 Table Multivariate logistic regression analyses for evaluating risk factors of non-primary tumor-bearing lobe metastases stratified by smoking status. (DOCX)

S3 Table Summary of dissected lymph nodes in 301 enrolled patients. (DOCX)

S4 Table Comparison of the resection cases with VATS and thoracotomy. (DOCX)

S5 Table Demographic information of enrolled patients and excluded patients with missing pathology reports. (DOCX)

S6 Table Summary of the patient with or without preoperative PET-CT exam in terms of NTBL compositions. (DOCX)

S7 Table Summary of adenocarcinoma subtypes. (DOCX)

S8 Table Summary of histological “others” in Table 4. (DOCX)
