# Supplementary material for: Risk factors of lobar lymph node metastases in non-primary tumor-bearing lobes among the patients of non-small-cell lung cancer
Source: PLoS One. 2020 Sep 17;15(9):e0239281. doi: 10.1371/journal.pone.0239281 (PMC7498110; doi:10.1371/journal.pone.0239281)
Supplement: S1 Table — (DOCX) [file pone.0239281.s001.docx]

| **Supplemental Table 1.**  Clinical staging information among NSCLC patients stratified by NTBL status | | | | | |
| --- | --- | --- | --- | --- | --- |
| TNM System | NTBL (-)  (N = 263) | | NTBL (+)  (N = 38) | | *P* value |
|  | N | % | N | % |  |
| T stage |  |  |  |  |  |
| T1 | 121 | 46.01 | 16 | 42.11 | 0.240 |
| T2 | 81 | 30.80 | 15 | 39.47 |  |
| T3 | 39 | 14.83 | 2 | 5.26 |  |
| T4 | 22 | 8.37 | 5 | 13.16 |  |
| N stage |  |  |  |  |  |
| N0 | 160 | 60.84 | 12 | 31.58 | 0.003 |
| N1 | 33 | 12.55 | 8 | 21.05 |  |
| N2 | 63 | 23.95 | 15 | 39.47 |  |
| N3 | 7 | 2.66 | 3 | 7.89 |  |
| M stage |  |  |  |  |  |
| M0 | 248 | 94.30 | 37 | 97.37 | 0.690 |
| M1 | 15 | 5.70 | 1 | 2.63 |  |
