# Supplementary material for: Risk factors of lobar lymph node metastases in non-primary tumor-bearing lobes among the patients of non-small-cell lung cancer
Source: PLoS One. 2020 Sep 17;15(9):e0239281. doi: 10.1371/journal.pone.0239281 (PMC7498110; doi:10.1371/journal.pone.0239281)
Supplement: S2 Table — (DOCX) [file pone.0239281.s002.docx]

| **Supplemental Table 2** Multivariate logistic regression analyses for evaluating risk factors of non-primary tumor-bearing lobe metastases stratified by smoking status | | | | | | | | | | | |
| --- | --- | --- | --- | --- | --- | --- | --- | --- | --- | --- | --- |
| Characteristics | Smokers | | | | |  | Non-smokers | | | | |
|  | NTBL (-) | NTBL (+) | Adjusted OR* | 95% CI | *P* value |  | NTBL (-) | NTBL (+) | Adjusted OR* | 95% CI | *P* value |
| Location |  |  |  |  |  |  |  |  |  |  |  |
| LLL | 24 | 5 | 1.00 |  |  |  | 14 | 6 | 1.00 |  |  |
| LUL | 30 | 0 | NA | NA | NA |  | 17 | 2 | 0.28 | 0.05 - 1.58 | 0.15 |
| RLL | 44 | 4 | 0.44 | 0.11-1.78 | 0.25 |  | 30 | 8 | 0.62 | 0.18 - 2.14 | 0.45 |
| RML | 8 | 2 | 1.20 | 0.19-7.44 | 0.85 |  | 8 | 2 | 0.58 | 0.09 - 3.60 | 0.56 |
| RUL | 51 | 3 | 0.28 | 0.06-1.28 | 0.10 |  | 37 | 6 | 0.38 | 0.10 - 1.37 | 0.14 |
| Location - 1 |  |  |  |  |  |  |  |  |  |  |  |
| RLL/RML/RUL (right) | 103 | 9 | 1.00 |  |  |  | 75 | 16 | 1.00 |  |  |
| LLL/LUL (left) | 54 | 5 | 1.06 | 0.34-3.32 | 0.92 |  | 31 | 8 | 1.21 | 0.47-3.12 | 0.69 |
| Location - 2 |  |  |  |  |  |  |  |  |  |  |  |
| LUL/RUL (upper) | 81 | 3 | 1.00 |  |  |  | 54 | 8 | 1.00 |  |  |
| LLL/RLL/RML (lower) | 76 | 11 | 3.91 | 1.05-14.55 | 0.04 |  | 52 | 16 | 2.08 | 0.82-5.27 | 0.12 |
| Tumor size |  |  |  |  |  |  |  |  |  |  |  |
| ≤3cm | 53 | 3 | 1.00 |  |  |  | 55 | 7 | 1.00 |  |  |
| >3cm and ≤5cm | 55 | 7 | 2.25 | 0.55-9.16 | 0.26 |  | 35 | 8 | 1.80 | 0.60-5.39 | 0.30 |
| >5cm and ≤7cm | 31 | 3 | 1.71 | 0.33-9.00 | 0.53 |  | 14 | 7 | 3.93 | 1.18-13.05 | 0.03 |
| >7cm | 18 | 1 | 0.98 | 0.10-10.04 | 0.99 |  | 2 | 2 | 7.86 | 0.95-64.93 | 0.06 |
| Trend |  |  |  |  |  |  |  |  |  |  |  |
| Lymph node metastases |  |  |  |  |  |  |  |  |  |  |  |
| TBL lobar |  |  |  |  |  |  |  |  |  |  |  |
| No | 85 | 2 | 1.00 |  |  |  | 75 | 2 | 1.00 |  |  |
| Yes | 72 | 12 | 7.08 | 1.54-32.70 | 0.01 |  | 31 | 22 | 26.61 | 5.90-120.09 | ＜0.001 |
| Interlobar/hilar |  |  |  |  |  |  |  |  |  |  |  |
| No | 134 | 5 | 1.00 |  |  |  | 96 | 7 | 1.00 |  |  |
| Yes | 23 | 9 | 10.49 | 3.23-34.11 | ＜0.001 |  | 10 | 17 | 23.31 | 7.80-69.69 | ＜0.001 |
| Mediastinal |  |  |  |  |  |  |  |  |  |  |  |
| No | 104 | 0 | 1.00 |  |  |  | 78 | 4 | 1.00 |  |  |
| Yes | 53 | 14 | NA | NA | NA |  | 28 | 20 | 13.93 | 4.38-44.30 | ＜0.001 |
| Multi-station mediastinal |  |  |  |  |  |  |  |  |  |  |  |
| No | 144 | 3 | 1.00 |  |  |  | 96 | 10 | 1.00 |  |  |
| Yes | 13 | 11 | 40.62 | 10.05-164.23 | ＜0.001 |  | 10 | 14 | 13.44 | 4.75-38.05 | ＜0.001 |
| VPI |  |  |  |  |  |  |  |  |  |  |  |
| No | 112 | 5 | 1.00 |  |  |  | 80 | 15 | 1.00 |  |  |
| Yes | 45 | 9 | 4.48 | 1.42-14.10 | 0.01 |  | 26 | 9 | 1.85 | 0.72-4.71 | 0.20 |
| LVI |  |  |  |  |  |  |  |  |  |  |  |
| No | 119 | 8 | 1.00 |  |  |  | 91 | 17 | 1.00 |  |  |
| Yes | 38 | 6 | 2.35 | 0.77-7.20 | 0.14 |  | 15 | 7 | 2.50 | 0.89-7.04 | 0.08 |
| AD, adenocarcinoma; LLL, left lower lobe; LUL, left upper lobe; LVI, lymph avascular invasion; NTBL, non-primary tumor-bearing lobe; RLL, right lower lobe; RML, right middle lobe; RUL, right upper lobe; TBL, tumor-bearing lobe; SCC, squamous cell carcinoma; VPI, visceral pleural invasion.  *: adjusted by the smoking history.  ^#^Others including large cell carcinoma, adenosquamous carcinoma, atypical carcinoid, carcinoma of salivary gland, etc. | | | | | | | | | | | |
