# Supplementary material for: Risk factors of lobar lymph node metastases in non-primary tumor-bearing lobes among the patients of non-small-cell lung cancer
Source: PLoS One. 2020 Sep 17;15(9):e0239281. doi: 10.1371/journal.pone.0239281 (PMC7498110; doi:10.1371/journal.pone.0239281)
Supplement: S3 Table — (DOCX) [file pone.0239281.s003.docx]

**Supplementary Table 3**. Summary of dissected lymph nodes in 301 enrolled patients

| Category | Number of dissected lymph nodes | NTBL (-) | NTBL (+) |
| --- | --- | --- | --- |
| N1 region | Number range | 4-31 | 4-22 |
|  | Mean number | 9 | 10 |
|  | Average number | 10.1 | 11.1 |
| N2 region | Number range | 3-49 | 3-48 |
|  | Mean number | 14 | 14 |
|  | Average number | 14.0 | 8.4 |
| NTBL region | Number range | 1-11 | 1-9 |
|  | Mean number | 2 | 3 |
|  | Average number | 2.3 | 2.9 |
