# Supplementary material for: Risk factors of lobar lymph node metastases in non-primary tumor-bearing lobes among the patients of non-small-cell lung cancer
Source: PLoS One. 2020 Sep 17;15(9):e0239281. doi: 10.1371/journal.pone.0239281 (PMC7498110; doi:10.1371/journal.pone.0239281)
Supplement: S4 Table — (DOCX) [file pone.0239281.s004.docx]

**Supplementary Table 4**. Comparison of the resection cases with VATS and thoracotomy.

|  | NTBL (-) | NTBL (+) | *P* value |
| --- | --- | --- | --- |
| VATS | 51 | 8 | 0.530 |
| Thoracotomy | 212 | 30 |  |
