# Supplementary material for: Risk factors of lobar lymph node metastases in non-primary tumor-bearing lobes among the patients of non-small-cell lung cancer
Source: PLoS One. 2020 Sep 17;15(9):e0239281. doi: 10.1371/journal.pone.0239281 (PMC7498110; doi:10.1371/journal.pone.0239281)
Supplement: S5 Table — (DOCX) [file pone.0239281.s005.docx]

**Supplementary Table 5**. Demographic information of enrolled patients and excluded patients with missing pathology reports

|  | Enrolled patients  (N = 301) | Excluded patients  (N = 40) | *P* value |
| --- | --- | --- | --- |
| Females | 100 | 16 | 0.50 |
| Males | 201 | 24 |  |
| Age (mean) | 60.3 | 59.8 | 0.65 |
