# Supplementary material for: Risk factors of lobar lymph node metastases in non-primary tumor-bearing lobes among the patients of non-small-cell lung cancer
Source: PLoS One. 2020 Sep 17;15(9):e0239281. doi: 10.1371/journal.pone.0239281 (PMC7498110; doi:10.1371/journal.pone.0239281)
Supplement: S6 Table — (DOCX) [file pone.0239281.s006.docx]

| **Supplemental Table 6**. Summary of the patient with or without preoperative PET-CT exam in terms of NTBL compositions. | | | | | |
| --- | --- | --- | --- | --- | --- |
|  | NTBL (-)  (N = 263) | | NTBL (+)  (N = 38) | | P value |
|  | N | % | N | % |  |
| PET-CT |  |  |  |  |  |
| Yes | 57 | 21.7 | 4 | 10.5 | 0.167 |
| No | 206 | 78.3 | 34 | 89.5 |  |
