# Supplementary material for: Risk factors of lobar lymph node metastases in non-primary tumor-bearing lobes among the patients of non-small-cell lung cancer
Source: PLoS One. 2020 Sep 17;15(9):e0239281. doi: 10.1371/journal.pone.0239281 (PMC7498110; doi:10.1371/journal.pone.0239281)
Supplement: S7 Table — (DOCX) [file pone.0239281.s007.docx]

**Supplementary Table 7**. Summary of adenocarcinoma subtypes

|  | NTBL (-) | NTBL (+) |
| --- | --- | --- |
| Acinar | 36 (13.7%) | 10 (26.3%) |
| Papillar | 7 (2.66%) | 0 (0%) |
| Micropapillar | 3 (1.14%) | 0 (0%) |
| Solid | 8 (3.04%) | 2 (5.26%) |
| Mucinous | 3 (1.14%) | 0 (0%) |
