# Supplementary material for: Risk factors of lobar lymph node metastases in non-primary tumor-bearing lobes among the patients of non-small-cell lung cancer
Source: PLoS One. 2020 Sep 17;15(9):e0239281. doi: 10.1371/journal.pone.0239281 (PMC7498110; doi:10.1371/journal.pone.0239281)
Supplement: S8 Table — (DOCX) [file pone.0239281.s008.docx]

**Supplementary Table 8**. Summary of histological “others” in Table 4.

|  | NTBL (-) | NTBL (+) |
| --- | --- | --- |
| Large cell carcinoma | 21 (7.98%) | 3 (7.89%) |
| Adenosquamous cell carcinoma | 5 (1.90%) | 0 (0%) |
| Others* | 6 (2.28%) | 0 (0%) |
| Total | 32 (12.2%) | 3 (7.89%) |

*, atypical carcinoid, salivary gland-derived, and sarcomatoid carcinoma
